# Raytracing Modelling of Infrared Light Management Using Molybdenum Disulfide (MoS_2_) as a Back-Reflector Layer in a Silicon Heterojunction Solar Cell (SHJ)

**DOI:** 10.3390/ma15145024

**Published:** 2022-07-19

**Authors:** Mohammed Islam Elsmani, Noshin Fatima, Ignacio Torres, Susana Fernández, Michael Paul A. Jallorina, Puvaneswaran Chelvanathan, Ahmad Rujhan Mohd Rais, Mohd Norizam Md Daud, Sharifah Nurain Syed Nasir, Suhaila Sepeai, Norasikin Ahmad Ludin, Mohd Asri Mat Teridi, Kamaruzzaman Sopian, Mohd Adib Ibrahim

**Affiliations:** 1 Solar Energy Research Institute (SERI), Universiti Kebangsaan Malaysia, Bangi 43600, Selangor, Malaysia; noshinfatima1990@gmail.com (N.F.); cpuvaneswaran@ukm.edu.my (P.C.); rujhanrais92@gmail.com (A.R.M.R.); mohdnorizam@ukm.edu.my (M.N.M.D.); sharifahnurain@ukm.edu.my (S.N.S.N.); suhailas@ukm.edu.my (S.S.); sheekeen@ukm.edu.my (N.A.L.); asri@ukm.edu.my (M.A.M.T.); ksopian@ukm.edu.my (K.S.); 2 Departamento de Energías Renovables, CIEMAT, 28040 Madrid, Spain; ignacio.torres@ciemat.es (I.T.); susanamaria.fernandez@ciemat.es (S.F.); 3 Information Device Science Laboratory, Nara Institute of Science and Technology (NAIST), 8916-5 Takayama-cho, Ikoma 630-0192, Japan; michael.jallorina.mf8@ms.naist.jp; 4 NAIST-École Polytechnique International Collaborative Laboratory, Nara Institute of Science and Technology (NAIST), 8916-5 Takayama-cho, Ikoma 630-0192, Japan; 5 School of Physics, Universiti Sains Malaysia, Gelugor 11800, Penang, Malaysia

**Keywords:** computer simulations, dimensionality reduction, light trapping, photovoltaic cells, raytracing, thin films

## Abstract

The silicon heterojunction solar cell (SHJ) is considered the dominant state-of-the-art silicon solar cell technology due to its excellent passivation quality and high efficiency. However, SHJ’s light management performance is limited by its narrow optical absorption in long-wave near-infrared (NIR) due to the front, and back tin-doped indium oxide (ITO) layer’s free carrier absorption and reflection losses. Despite the light-trapping efficiency (LTE) schemes adopted by SHJ in terms of back surface texturing, the previous investigations highlighted the ITO layer as a reason for an essential long-wavelength light loss mechanism in SHJ solar cells. In this study, we propose the use of Molybdenum disulfide (MoS_2_) as a way of improving back-reflection in SHJ. The text presents simulations of the optical response in the backside of the SHJ applying the Monte-Carlo raytracing method with a web-based Sunsolve high-precision raytracing tool. The solar cells’ electrical parameters were also resolved using the standard electrical equivalent circuit model provided by Sunsolve. The proposed structure geometry slightly improved the SHJ cell optical current density by ~0.37% (rel.), and hence efficiency (*η*) by about 0.4% (rel.). The SHJ cell efficiency improved by 21.68% after applying thinner back ITO of about 30 nm overlayed on ~1 nm MoS_2_. The efficiency improvement following the application of MoS_2_ is tentatively attributed to the increased NIR absorption in the silicon bulk due to the light constructive interface with the backside components, namely silver (Ag) and ITO. Study outcomes showed that improved SHJ efficiency could be further optimized by addressing front cell components, mainly front ITO and MoS_2_ contact engineering.

## 1. Introduction

With the continuously increasing global energy demand, solar cells are projected to be one of the most important renewable resources to offset depletable conventional resources and reduce the likelihood of irreversible damage to the global environment. Presently, crystalline silicon solar cell holds a share market of about 95%, leaving the remaining 5% to thin-film solar cell technologies [1,2,3,4]. Despite the widespread use of advanced silicon solar cells (c-Si) with cutting-edge passivating and light management techniques, the near-infrared light response still has room for improvement. Nasir et al. [5] showed that the weak absorption of near-infrared light spectrum range (NIR) (900–1000 nm) is mainly due to the indirect bandgap of silicon at long wavelengths (i.e., bandgap cut-off wavelength 1200 nm). Moreover, back-reflector parasitic absorption, unwanted reflection, free carrier absorption, and plasmonic effects are the primary loss mechanisms in back-reflection layers [6,7]. Therefore, the condition for a perfect solar cell that may resemble an ideal diode is external luminescence, which balances the internal luminescence. A perfect back-reflecting mirror or a back reflector (BR) with ideally high reflectivity and low loss medium is required to enhance light trapping and improve the *V_oc_* [6]. 

In general, silicon solar cell back-reflection loss was systematically treated in many pieces of literature with the hopes of addressing back-reflection layer loss as a means for countering the escaped front, escape back and parasitically absorbed light loss using the perfect Lambertian mirror (i.e., BR = 1) [8,9,10]. In contrast, throughout the past decades, continuous and sustained development of light trapping technologies has remarkably been improved, reaching today’s hi-tech silicon heterojunction solar cell (SHJ) technology with an efficiency of over 26% in the interdigitated back contacts (IBC) configuration [11,12]. The solar cell’s high efficiency of SHJ is attributed to the high open-circuit voltage (*V_oc_*) thanks to the microstructural hydrogenated amorphous silicon layer (a-Si:H). However, NIR loss (over 1000 nm) in the back-reflection contact layers is not trivial [13]. Holman and co-workers [13] demonstrated that among SHJs, NIR (over 1000 nm) experience a loss with wafer texturing trapping scheme, which is weak or barely counted due to the complex contribution of the front and back transmitting conductive oxides (TCOs), namely indium doped tin oxide (ITO). 

In the SHJ case where transparent conductive oxides (TCOs) are essentially intended to play a synergetic role of high light transparency (i.e., also act as ARC, allowing the photon coupling to the solar cell) and electronically transfer the generated photo carriers to the external circuit [13]. However, as an example of ITO, TCO’s performance limit is a tradeoff between free carrier absorption (FCA) and high sheet resistivity. Further, the sole relief for the ITO optimum condition parameterization variation is accomplished with high mobility ITO [13,14]. Furthermore, the implication of NIR loss in the back-reflector contact layers is extended to become a prominent factor in the perovskite-silicon tandem (PSC-Si) solar cells. Bush et al., showed that NIR loss represents ~17% in the backside contact of two-terminal (2T) PSC-Si tandem solar cells. While back-reflectance loss amounts to ~10%, the parasitic NIR loss of ~3.3 mA is due to low light trapping at the back-reflector and losses at the front and back ITO contact of the SHJ solar cell [15,16]. Despite the half-spectrum configuration in four-terminal (4T), ITO parasitic absorption and reflection burdened the 4T tandem configuration due to the multiple TCO layers required. Whereas for other tandem configurations (i.e., 2T and 3T), light losses are less pronounced than the 4T tandem as there are fewer TCO layers. Reduction of NIR long-wavelength loss at the back-reflected contact is an active area of research, with approaches such as the adoption of highly transparent and high mobility TCOs [15], rear-side chemical polishing (RSCP) to reduce pyramid texturing and recombination mechanisms [17] or complex and expensive methods, including photonic crystal-based distributed Bragg reflector (DBR) [18,19]. 

Transition Metal Di-chalcogenide (TMDC) material Molybdenum disulfide (MoS_2_) is widely investigated in photocatalytic hydrogen production owing to its unique optical and electrical properties [20,21,22,23]. Thinner layers of MoS_2_, like most of the TMDCs, are shown to possess a higher band gap of ~1.8 eV—for monolayer—due to band spin-orbit coupling and less defective interface due to the weak interlayer van der Waals (vdW) interactions, which may make it suitable candidate material for heterostructure solar cell applications. Moreover, MoS_2_ shows considerable theoretical carrier mobility of ~200 cm^2^ V^−1^ s^−1^ for monolayers and ~500 cm^2^ V^−1^ s^−1^ for multi-layer [24]. Similarly, MoS_2_ is suggested as an active layer in solar cells due to its high absorption coefficient in the wide visible light range and the enhanced induced drift electrical field [25]. In a comprehensive review, Das et al. [24] showed that MoS_2_ was widely reported as an active layer in heterojunction p-silicon and n-silicon configurations. The MoS_2_ solar cell efficiency with varying MoS_2_ thickness has proved that the introduction of MoS_2_ has significantly improved graphene-silicon solar cell efficiency from 0.91% (abs.) in monolayer configuration to 11.1% for 9 nm MoS_2_ thickness [24]. Various relevant MoS_2_ solar cell configurations can be found in [26,27,28]. 

Although MoS_2_ is a promising candidate for solar cells, the absorptivity of the MoS_2_ Monolayer as a window layer may amount to ~5–10% of incident light [24]. This loss is because the window layer reportedly exhibited some limitations due to a considerably high reflectivity, which could be beneficial for back reflection in the NIR long-wavelength [29,30,31]. Iqbal et al. [32] showed the possibility of using MoS_2_/Mo as a composite back-reflection layer in the dye-sensitized solar cell (DSSC). The method seems promising thanks to the reflected light from the counter electrode (CE) towards TiO_2_, which results in more light being trapped by the solar cell. 

**Figure 1:**
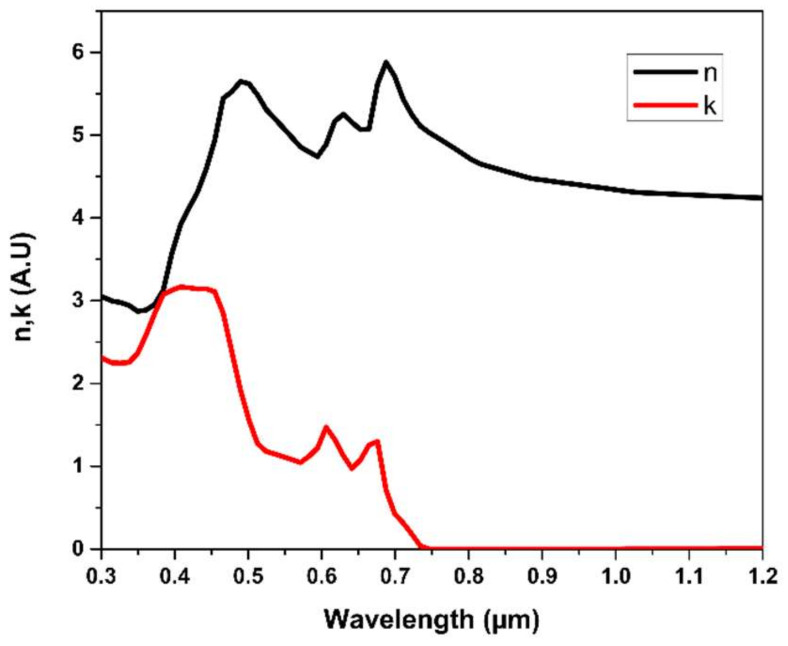
The optical characteristic of MoS2 n and k-value was reprinted with permission from Ref [34]. https://refractiveindex.info/about (accessed on 14 July 2021).

Theoretically, one of the main requirements in a solar cell’s back reflection is the Lambertian scatterer, which satisfies the light path increment and results in light absorption enhancement [7]. The improvement can be achieved through white back-reflective material since light path length can be increased by 2n^2^, where n is the active material’s refractive index [33,34,35]. For MoS_2_ as back-reflector material, in addition to its relatively high reflectivity in the NIR region inherited from the real refractive index (see Figure 1), MoS_2_ uniquely possesses a low-light-absorption profile due to its low extinction coefficient (k) in the long wavelength, which both (i.e., high real refractive index and low absorptive coefficient) makes it a suitable candidate for near-infrared (NIR) > 700 nm [31,34]. The low absorption can be observed in the long-wavelength of bulky MoS_2_ in Figure 1 [36], where k (i.e., imaginary refractive index) asymptotically vanishes. The k value measures electromagnetic wave dampening related to (Equation (1)) absorption coefficient (α is the light absorbed in the media). A similar back-reflector material selection approach can be found in Refs. [37,38]. Furthermore, MoS_2_ exhibits considerable carrier mobility, which may positively enhance solar cell carrier transport and reduce solar cell loss [24].

(1) α=4πk/λ

where *α* is the absorption coefficient, *k* is the extinction coefficient, and *λ* is the wavelength.

Therefore, in-depth investigation and prediction with validated novel technology simulation are required before performing the experimental analysis. In this contribution, MoS_2_ optical potentiality as a back-reflector layer in photo-current light current is investigated and quantified in the silicon heterojunction solar cell (SHJ) using the web-based Sunsolve^TM^ high precision raytracing tool from PV lighthouse [39], which is explained in the method section. MoS_2_ layer optical data (real and imaginary) refractive indices were imported from MoS_2_ in Reference [34]. The results and discussion section evaluate the application of MoS_2_ as a back-reflected layer for the solar cell in terms of photo-current density and absorption profile. 

## 2. Methodology

This paper assumes the accuracy of the imported data and applicability of MoS_2_ thickness referred to within this article (see Figure 1) [36]. Another monolayer (~<1 nm) and a few layers of MoS_2_ were taken from references [40,41], respectively, and applied for each case as required. Web application-based Sunsolve^TM^ simulation tool evaluates the optical response in loss (gain) in the various solar cell regions under standard illumination sources. Photo-generated current generated inside the solar cell and collected by the p–n junction (*J_L_*) is calculated following (Equation (2)) employing a combination of a complex algorithm of Monte-Carlo’s ray-traced algorithm method and thin-film optics [42]:

(2) $$J_{L}=\int_{0}^{\infty }J_{ph}\;\left(\lambda \right)\;A\left(\lambda \right)\;\eta \left(\lambda \right)d\lambda$$

where *J_ph_*(λ) is the incident photon current as determined from photonic flux incident light density, *A(λ)* is the fraction of the incident light that becomes absorbed by the active region of the solar cell as determined by raytracing, and *η(λ)* is the collection efficiency within that active region. The detail of all parameters is followed as per the literature [39].

The adopted Monte Carlo method by Sunsolve is commonly used for coherent and incoherent light sources based on the probability distributions of individual traced rays. The optimized version of Monte-Carlo’s method can treat a high number of rays (in this manuscript ~5E5 per simulation run), improving the scattered rays phase and thus reducing the computational cost. 

All simulation models fixed the applied solar spectrum at AM 1.5 G (100 mW/cm^2^) and a temperature of 25 °C. Solar cells’ morphology was assumed to follow the standard SSP (crystalline silicon screen-print Aluminum back surface field (Al-BSF) mono-facial solar cell) and SHJ cell architecture defined in the standard web-application Sunsolve model. Solar cell contact and texturing parameters are briefed in Table 1. More information can be found on the Sunsolve-PVlighthouse website [39].

**Figure 2:**
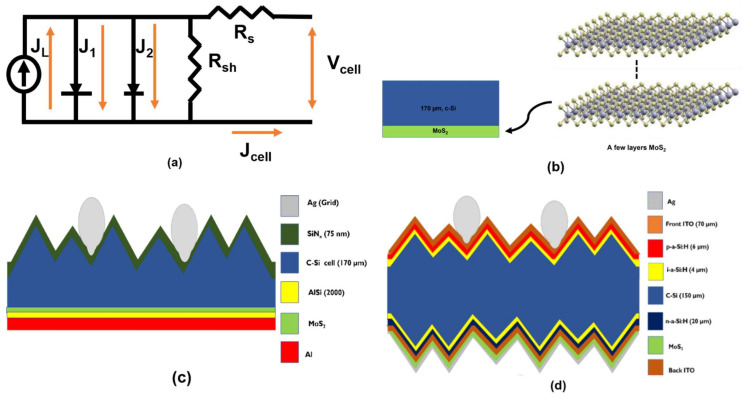
(**a**) Sunsolve electrical equivalent circuit model, (**b**) silicon wafer along with MoS_2_ few layers at the backside, (**c**) SSP solar cell and (**d**) SHJ solar cell. (All figures not to scale).

After fixing SHJ solar cell resistance parameters throughout the simulation models, other solar cell parameters such as open-circuit voltage (*V_oc_*) and fill factor (*FF*) are based on the equivalent circuit model obtained from Reference [39] and shown in Figure 2a. Figure 2 also shows the different structures simulated in this work: a silicon wafer with a few layers of MoS_2_ (b), an SSP silicon solar cell (c) and an SHJ solar cell. In the case of SHJ solar cells, we considered different structures according to the conditions briefed in Table 2.

## 3. Results

To assess the effect of MoS_2_ on the photo-current density, we initially focused on the computational simulation of the structure shown in Figure 2b, i.e., a flat, bare silicon wafer (170 µm) with varying thickness of MoS_2_, before proceeding to the simulation of complete solar cells. 

### 3.1. Photo-Current Density Simulation

**Figure 3:**
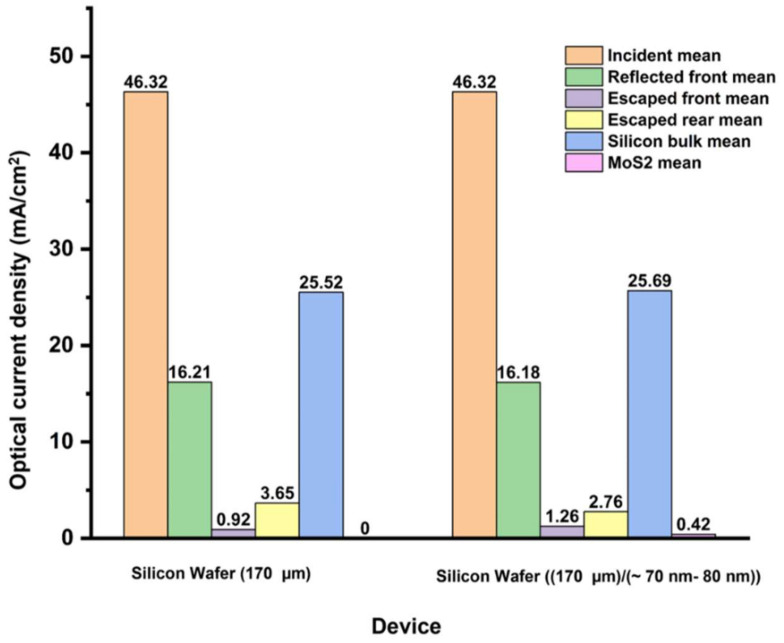
Photo-current density loss was calculated for a silicon wafer and a silicon wafer/MoS_2_ structure.

By varying the MoS_2_ layer thickness from 1 nm to 100 nm, the photo-current density improved in the silicon bulk by 0.7% at 25.69 mA/cm^2^. Such slight current improvement is obtained at an optimum MoS_2_ thickness of 70–80 nm. Figure 3 summarizes the current density loss in the silicon wafer-MoS_2_ model. Remarkably, following MoS_2_ layer application, the total improved difference of the absorbed current density in the silicon bulk was 0.92 mA/cm^2^ showing that it had benefited from the components of the reduced escaped rear and reflected front currents, respectively. On the other hand, the MoS_2_ thick film as a rear non-contact interface absorbed 0.42 mA/cm^2,^ which is attributed to the increased light absorption, as mentioned in Reference [41]. A room for improvement could be even further when obtained via front surface texturing optimization and the application of back metallization. 

**Figure 4:**
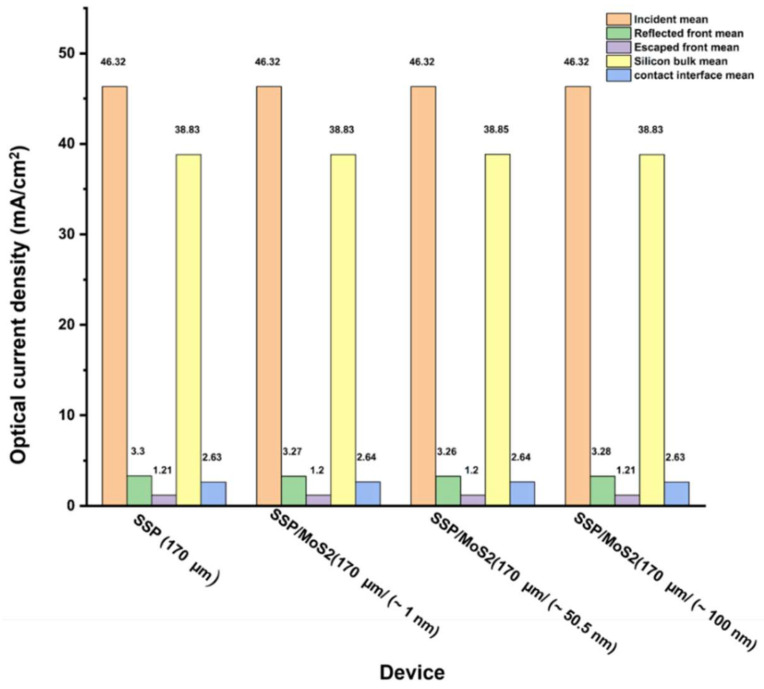
Photo-current density loss was calculated for the SSP solar cell and the SSP solar cell/MoS_2_ structure with various MoS_2_ thicknesses.

Following the initial MoS_2_ layer variation in bare wafer silicon, the SSP solar cell was simulated using MoS_2_ as a back-reflector layer (see Figure 2c). Figure 4 summarizes the current density obtained for this structure for MoS_2_ thicknesses between 1 nm and 100 nm. As can be seen, the application of front surface texturing and back metallization reduced escaped front light loss compared to the bare silicon wafer.

The best solar cell bulk current (38.85 mA/cm^2^) was obtained at MoS_2_ thickness ~50 nm. Interestingly, from a thin-film optics perspective, this MoS_2_ thickness value nearly corroborates the ideal destructive interference theoretical value (~50 nm) characterized by (Equation (3)) at a wavelength of 1000 nm and refractive index absolute values ranging between ~4.5–5.0 (refer to Figure 1). The equation of optical thickness d (nm) is determined by:

(3) d=λ/4n

where *λ* is the wavelength in (nm), and *n* is the refractive index. 

While the non-contact SSP rear solar cells (i.e., MoS_2_) yielded ‘0’ mA/cm^2^, we summarize that the slight improvement could be attributed to the back side MoS_2_/AlSi alloy back surface field constructive interference and reduced front reflection. The MoS_2_ influence on the latter is unclear to us. We attribute the extra loss in light current in the SSP/MoS_2_ (~50.5 nm) to the AlSi alloy back surface field contact-induced optical contact loss, which takes place regardless of MoS_2_ layer thickness quenched the *J_L_* enhancement in silicon solar cells.

The electrical cell parameters were calculated using the equivalent circuit model in Figure 2a by Sunsolve. The simulated electrical cell parameters are depicted in Table 3. It is observed that the slight loss of *V_oc_* and *FF* in SSP/MoS_2_, such that the addition of MoS_2_ in the back contact of SSP did not improve the overall cell efficiency.

Since SHJ (see Figure 2d) nowadays represents an integral part of the highly efficient perovskite silicon tandem solar cell and is projected to have >15% PV market share by 2030, the requirements for overcoming or reducing back-reflection loss of the bottom cell are tangible [43]. Thus, the final simulation was conducted on SHJ solar cells applying MoS_2_ as a back-reflection layer in the configurations (D1, D2, D3 and D4) referenced in Table 2.

**Figure 5:**
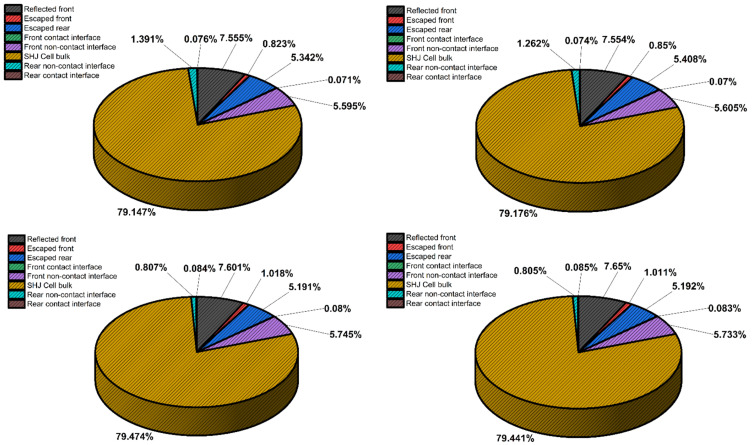
Pie charts represent optical current density in SHJ cell using MoS_2_ as back-reflector layer for various structure: (**a**) SHJ; (**b**) SHJ/MoS_2_ (1 nm)/ITO (70 nm)/Ag; (**c**) SHJ/MoS_2_ (1nm)/ITO (30 nm)/Ag; and (**d**) SHJ/ITO (30 nm)/MoS_2_ (1 nm)/Ag.

Initial simulations with the application of bulky and few layers of MoS_2_ (other than a monolayer of MoS_2_) on SHJ had worsened the performance regardless of MoS_2_ layer thickness. The pie chart (rounded to the nearest significant digit) of Figure 5a depicts the SHJ light density distribution percentage used as a reference for the successive simulations. Figure 5b shows Device ‘D2’ where MoS_2_ combined with higher ITO thickness increased rear non-contact optical current density loss by about 0.13%. However, in ‘D3’, with a further reduction of the ITO layer down to 30 nm thickness, the application of MoS_2_ on SHJ in MoS_2_/ITO/Ag has started to become promising mainly due to the reduction of rear non-contact interface and escape rear loss at 0.81% and 5.19% respectively (see Figure 5c). In this structure, it was observed that the increment of the escape front is comparable to the Silicon/MoS_2_ trend in Figure 3 despite front texturing, which is unclear to us. It can be elucidated that the provision of MoS_2_ along thinner ITO had increased the light path. Therefore, it is required to optimize front texturing/front light further trapping to better trap reflected light in the solar cell active layer.

**Figure 6:**
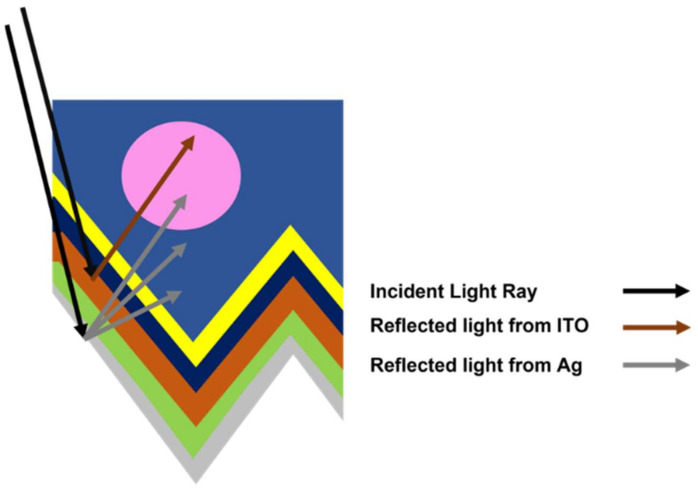
A NIR light rays components constructive interference has conceptually drawn in the pink circle.

Finally, MoS_2_ was interchanged with ITO in the simulation scenario, device ‘D4’, depicted in Figure 5d. The result showed that solar cell current density yield (‘D4’~36.70 mA/cm^2^) had improved SHJ solar cell efficiency by almost 0.04% rel.), as shown in Table 4. This improvement is due to the reduction in the rear and rear’s non-contact light interface loss, probably due to the constructive light interference with the Ag electrode, as shown in Figure 6. The optical current improvement in ‘D4’ slightly increased the *V_oc_*, which resulted in higher overall efficiency. Nevertheless, compared with reference SHJ ‘D1’ Figure 5a, the limiting factor for solar cell configuration in ‘D4’ is the loss in the front part of the solar cell manifested in front non-contact, front contact, escape front, and reflected front components. 

**Figure 7:**
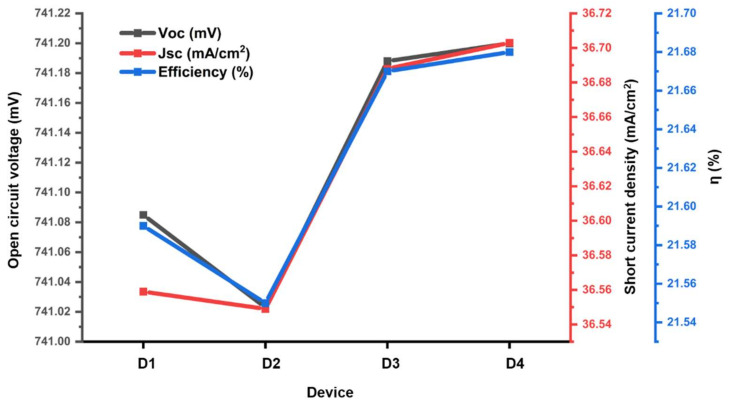
*J_sc_*, *V_oc_* and efficiency trend in All SHJ device configurations in Table 2.

Table 4 and Figure 7 show that the *J_sc_* enhancement in ‘D4’ has resulted in *V_oc_*, hence, the overall efficiency [44]. However, all the MoS_2_-based devices undergo lower *FF* than the reference SHJ, which requires more optimization in the SHJ device series resistance.

In order to further understand the optical physics of the improved *J_sc_* in Table 4, solar cell structures, external quantum efficiency, and reflectance profile for those mentioned above SHJ solar cells structures are referred to in the following sub-section

### 3.2. EQE Profile

**Figure 8:**
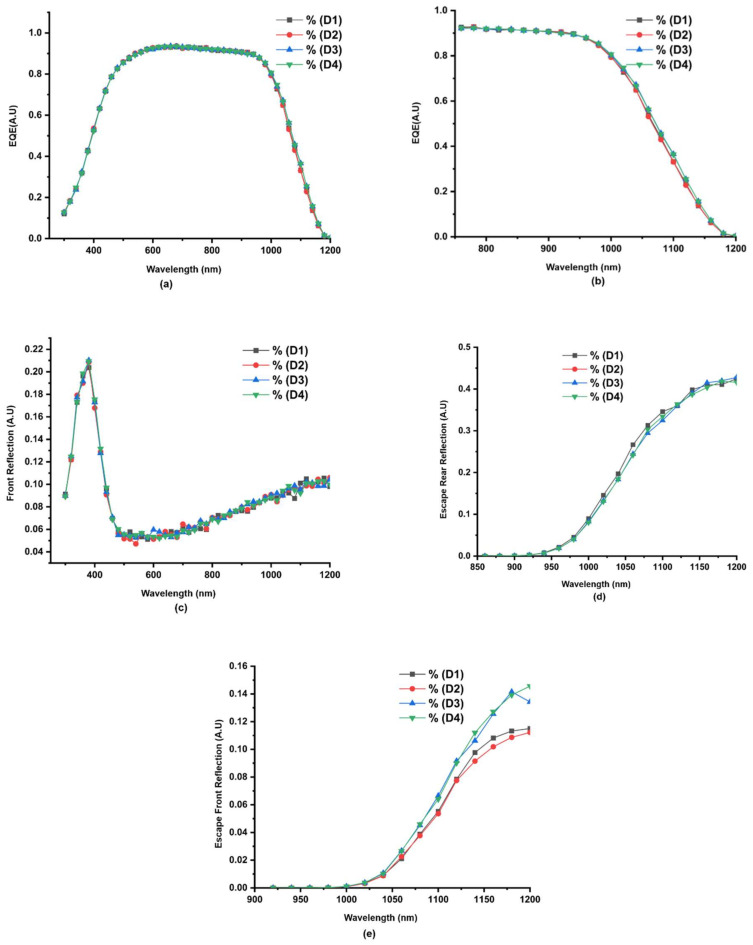
Graph of EQE vs wavelength and various losses in D1, D2, D3 and D4: (**a**) EQE and Reflectance for various SHJ structures (D1, D2, D3 and D4); (**b**) EQE in NIR; (**c**) Front reflection; (**d**) Escape front reflection; and (**e**) escape rear reflection.

The interest of this article is to explore MoS_2_ as a back-reflection layer in SHJ solar cells. Thus, external conversion efficiency (EQE) in Figure 8a and EQE in NIR region in Figure 8b, respectively, are conveniently required to understand the obtained SHJ’s power conversion efficiency (PCE) improvement shown in Table 4.

Figure 8c–e depicts reflectance of SHJ solar cells structure for front reflection, escape front reflection, and finally, escape rear reflection. Interestingly, in Figure 8b, EQE of thinner ITO (i.e., D4) is slightly more prominent in the long NIR (between black arrows) of about ‘0.14 mA/Cm^2^’, namely in the range of (970–1150 nm), than the rest of the ITO-based SHJ solar cell structures (D1, D2 and D3). It is likely attributed to the improved escape rear reflection (Figure 8d), which sustains our hypothesis of the back layers/MoS_2_ constructive light interference. On the other hand, thicker ITO seems to shift the escape rear reflectance towards a more extended wavelength red-shift (1040 nm), unlike thinner ITO, which tends to yield high reflectivity at lower wavelengths—blue shift. Such phenomena can be attributed to the thickness of ITO over the silicon wafer, making ITO behave as an anti-reflective layer. Zhen et al. [28] showed that the wavelength dependence reflectance of thicker ITO on the silicon wafer tends to shift towards a longer wavelength. Conversely, since ITO doping is considered fixed throughout the simulation models, the plasma resonance effect is assumed to be absent or less influenced by escape rear-reflectance [29]. In contrast, it can be speculated that high escape rear-reflectance may result from evanescence wave coupling between ITO and silver back contact as the rear ITO grows much thinner. 

Unfortunately, the implications of the ITO thinning application may electronically result in higher sheet resistivity (R_SH_), which may influence the electronic performance and counter the optical performance gained by the application of MoS_2_. In that regard, for SHJ in the full spectrum (300–1200 nm), the only relief for the rear back contact optical gain is to increase ITO mobility according to the R_SH_ = 1/(eNµt), where e is the elementary electron charge; N is the charge density; µ is the mobility; and t is the thickness, respectively [10]. However, in the case of the tandem-based SHJ half spectrum (i.e., PSC-Si at 700–1200 nm), the condition for mobility constraints might be applied only for the front ITO optimization instead of the rear ITO while keeping the rear ITO thinner.

In order to further explore NIR long wavelength loss minimization, future investigations may also require front ITO thickness optimization. Further optimization of front ITO may involve the state-of-the-art transparent conductive oxides (TCOs) and the graphene optimization process with more light transparent and higher mobility [30,45]. Furthermore, MoS_2_ contact engineering is highly required as the parasitic Schottky barrier and the effective material resistivity may impair the charge carrier extraction and the overall *FF* [46]. Nonetheless, the passivation evaluation is needed to assess the influence of MoS_2_ on SHJ open circuit voltage and *FF*. Such optimization will be detailed in future works.

To sum up, this work represents an endeavour toward exploring 2D materials for the backside of the solar cells so that we may be able to harness some gain in the NIR region in SHJ solar cells via the use of MoS_2_ materials.

## 4. Conclusions

This study sheds light on the TMDC material (MoS_2_) applied to the back of an SHJ solar cell. Following the initial investigation of MoS_2_ viability as a back-reflection layer on the bare silicon wafer and silicon solar cell, the application of thinner MoS_2_ (in the range of 1 nm) on a thin rear ITO ~30 nm as a back-reflector layer for the silicon solar cells were optically simulated on an SHJ solar cell. Remarkably, it was found that the application of MoS_2_ as a back-reflection layer optically improved SHJ solar cell efficacy using the solar cell equivalent circuit by a fraction of about 0.4% (rel.), possibly due to the improved short circuit current (*J_sc_*~0.14 mA/cm^2^) as a result of constructive light interference in the long-wave range, namely in the NIR. The *J_sc_* improvement induced *V_oc_* enhancement, resulting in a slight efficiency improvement following the application of Monolayer MoS_2_ on the back side of the SHJ. This enhancement in SHJ/MoS_2_ is based on thin ITO (~30 nm). However, further thinning of the rear ITO could be responsible for increased sheet resistance, the increased escape rear reflection and, thus, the increment of the evanescence wave coupling to the rear metal contact. Though this work shows fractional improvement in SHJ solar cell efficiency through MoS_2_ as a back-reflection layer, it may prove instrumental in the future design of solar cells with the high mobility front ITO optimization process alongside low resistance MoS_2_ contact engineering.

## Figures and Tables

**Table 1:** Electrical contacts and surface texturing of the SSP and SHJ solar cells simulation parameter.

Device	Metal Contact Parameters
SSP Front Electrode	Ag-Dupont PV19, resistivity: 2.6 × 10^−6^ Ω·cm. Grid (H: 15 µm × W: 45 µm). Finger pitch (0.15 cm). Finger spacing (~0.14 cm)
SSP Back Electrode	Al-Paste, resistivity: 5 × 10^−5^ Ω·cm. Full contact
SSP Front Texturing	Random upright pyramids (Angle: 52, H: 5 µm, W: 7.813 µm)
SHJ Front Electrodes	Custom Ag, resistivity 5.0 × 10^−6^ Ω·cm. Grid (H: 30 µm × W: 45 µm). Finger pitch (0.13 cm). Finger spacing (0.12 cm)
SHJ Back Electrodes	Custom Ag, resistivity 5.0 × 10^−6^ Ω·cm. Grid (H: 30 µm × W: 45 µm). Finger pitch (0.13 cm). Finger spacing (0.12 cm) or full contact electrode
SHJ Front and Back Texturing	Random upright pyramids (Angle: 52, H: 5 µm, W: 7.813 µm)

**Table 2:** Definition of the various SHJ structures considered in this work.

Device	Device Ref.
SHJ	D1
SHJ (150 µm)/MoS_2_ (~1 nm)/ITO (70 nm)	D2
SHJ (150 µm)/MoS_2_ (~1 nm)/ITO (30 nm)	D3
SHJ (150 µm)/ITO (30 nm)/MoS_2_ (~1 nm)	D4

**Table 3:** Photovoltaic parameters (*FF*, *V_oc_*, *J_sc_* and *η*) were calculated for the SSP solar cell and SSP/MoS_2_ structure using Sunsolve software.

Device	*FF* (%)	*V_oc_* (mV)	*J_sc_* (mA/cm2)	Efficiency-*η* (%)
SSP (170 µm)	74.80	630.32	38.81	18.30
SSP/MoS_2_ (170 µm/(~1 nm))	74.72	630.29	38.82	18.15
SSP/MoS_2_ (170 µm/(~50.5 nm))	74.71	630.30	38.84	18.29
SSP/MoS_2_ (170 µm/(~100 nm))	74.72	630.29	38.82	18.28

**Table 4:** Summary result of SHJ solar cell/MoS_2_ electrical parameters.

Device	*FF* (%)	*V_oc_* (mV)	*J_sc_* (mA/cm2)	Efficiency-*η* (%)
SHJ	79.71	741.09	36.56	21.60
SHJ/MoS_2_/ITO(170 µm/ (~1 nm)/70 nm)	79.59	741.02	36.55	21.56
SHJ/MoS_2_/ITO(170 µm/ (~1 nm nm)/30 nm)	79.71	741.19	36.69	21.68
SHJ/ITO/MoS_2_(170 µm/ (~30 nm)/1 nm)	79.71	741.20	36.70	21.68
